# Post-trial perceptions of a symptom-based TB screening intervention in South Africa: implementation insights and future directions for TB preventive healthcare services

**DOI:** 10.1186/s12912-021-00544-z

**Published:** 2021-02-08

**Authors:** Nicole Salazar-Austin, Minja Milovanovic, Nora S. West, Molefi Tladi, Grace Link Barnes, Ebrahim Variava, Neil Martinson, Richard E. Chaisson, Deanna Kerrigan

**Affiliations:** 1 Department of Pediatrics, Johns Hopkins University School of Medicine, 200 N. Wolfe Street Room 3147, Baltimore, MD 21287 USA; 2 Perinatal HIV Research Unit (PHRU), University of Witwatersrand, Johannesburg, South Africa; 3 Department of International Health, Johns Hopkins Bloomberg School of Public Health, Baltimore, MD USA; 4 Center for Tuberculosis Research, Johns Hopkins University School of Medicine, Baltimore, MD USA; 5 Department of Internal Medicine, Klerksdorp/Tshepong Hospital Complex, North West Province Department of Health, Klerksdorp, South Africa and University of the Witwatersrand, Johannesburg, South Africa; 6 Department of Prevention and Community Health, George Washington University Milken Institute School of Public Health, Washington, DC USA

## Abstract

**Background:**

Tuberculosis is a top-10 cause of under-5 mortality, despite policies promoting tuberculosis preventive therapy (TPT). We previously conducted a cluster randomized trial to evaluate the effectiveness of symptom-based versus tuberculin skin-based screening on child TPT uptake. Symptom-based screening did not improve TPT uptake and nearly two-thirds of child contacts were not identified or not linked to care. Here we qualitatively explored healthcare provider perceptions of factors that impacted TPT uptake among child contacts.

**Methods:**

Sixteen in-depth interviews were conducted with key informants including healthcare providers and administrators who participated in the trial in Matlosana, South Africa. The participants’ experience with symptom-based screening, study implementation strategies, and ongoing challenges with child contact identification and linkage to care were explored. Interviews were systematically coded and thematic content analysis was conducted.

**Results:**

Participants’ had mixed opinions about symptom-based screening and high acceptability of the study implementation strategies. A key barrier to optimizing child contact screening and evaluation was the supervision and training of community health workers.

**Conclusions:**

Symptom screening is a simple and effective strategy to evaluate child contacts, but additional pediatric training is needed to provide comfort with decision making. New clinic-based child contact files were highly valued by providers who continued to use them after trial completion. Future interventions to improve child contact management will need to address how to best utilize community health workers in identifying and linking child contacts to care.

**Trial registration:**

The results presented here were from research related to NCT03074799, retrospectively registered on 9 March 2017.

**Supplementary Information:**

The online version contains supplementary material available at 10.1186/s12912-021-00544-z.

## Background

Tuberculosis remains a top-10 cause of under-5 mortality worldwide, with over 200,000 estimated child TB deaths in 2018 [1, 2]. TB preventive therapy (TPT) reduces the risk of TB disease by more than 60% [3, 4]. Well-implemented pediatric contact investigation programs with preventive therapy may reduce TB-associated pediatric mortality by over 75% [5]. Yet despite long-standing recommendations, TB preventive therapy remains poorly implemented among household child TB contacts worldwide [2]. Only 27% of children exposed to TB in their homes received TPT in 2018 [2]. The UN held a high-level meeting (UNHLM) on TB and heads of states committed to improving TB prevention, including providing 4 million child and 20 million adult household TB contacts with TPT by 2022 [6].

South Africa carries a significant brunt of the global TB burden with an incidence of 520 cases per 100,000 persons [2]. They report up to 59% of child TB contacts under 5 years of age were evaluated and placed on TB preventive therapy in 2018 [2]. The Matlosana municipality is a semi-urban area within the Dr. Kenneth Kaunda district of North West Province, South Africa known to have high HIV and TB burdens. The HIV prevalence was 34% among pregnant women in 2017 and TB incidence was 690 per 100,000 persons in 2015 [7]. South Africa has boldly proposed to treat *all* household contacts of infectious TB index patients, irrespective of age, to address their TB epidemic. Understanding the barriers that prevent provision of TPT among child TB contacts is critical to developing responsive healthcare delivery models to reach the UNHLM targets.

The pediatric TB prevention continuum of care begins with identification of children through contact tracing, which is the enumeration of all household members of the TB index patient. Child TB contacts under 5 years of age are referred to the clinic for screening, and after assessment, initiation of either TPT or TB treatment. Follow up visits are scheduled for 6 months thereafter. In 2006, the World Health Organization recommended symptom-based evaluation without tuberculin skin testing (TST) for all child TB contacts, thereby simplifying the pediatric contact screening process [8]. South Africa followed suit in 2013 [9]. With a symptom-based approach, TPT can be initiated at the first clinic visit, irrespective of the TB infection status, for all asymptomatic TB-exposed children under 5 years of age. Symptomatic children require additional workup that may include a chest X-ray, a gastric aspirate and evaluation by a physician prior to initiation of either TB treatment or TPT. Despite this recommendation, uptake of symptom-based screening of child TB contacts was limited.

We conducted a cluster-randomized trial to evaluate the effectiveness of symptom-based screening on TPT uptake, as compared to TST-based screening under pragmatic conditions in Matlosana, South Africa from October 2015 to February 2017 [10]. The primary intervention was the introduction of symptom-based screening into eight South African primary health care clinics. The remaining eight clinics continued to provide TST-based screening and were trained in symptom-screening following the trial. To support implementation in all clinics, we also conducted initial and refresher training in either symptom-based or TST-based screening, provided supportive supervision, introduced a new clinic-based child contact medical record and register, and conducted audit and feedback sessions both to improve uptake of the medical record and to identify and address barriers along the child TB contact continuum of care.

The trial found that symptom-based screening did not improve the proportion of identified child TB contacts evaluated or initiated on TPT, compared with TST-based screening [10]. Symptom-based screening was conducted safely by nurses in community clinics with less than 1% of asymptomatic child TB contacts diagnosed with TB disease while taking TPT. Finally, heavy losses were found early in the care continuum with nearly two thirds of child TB contacts either not identified or not linked to clinic-based care. Few studies have previously explored healthcare worker perceptions of interventions to improve TPT uptake including symptom-based screening or the aforementioned implementation strategies used to support the intervention [11]. Likewise, few studies have assessed facilitators and barriers to identification of child TB contacts and linkage to care from the perspective of healthcare workers [11]. After the trial was completed, qualitative research methods were used to explore the nurses’ and administrators’ experiences with symptom-based screening, study implementation strategies, and ongoing challenges with child contact identification and linkage to facility-based care in order to inform wider roll out of symptom-based screening and develop future interventions to improve outcomes among child TB contacts in South Africa.

## Methods

### Study setting

From December 2017 to September 2018, we conducted 16 semi-structured, in-depth interviews 6 months after completion of a cluster-randomized trial [10]. At the time of data collection, child contact evaluation was performed by the focal TB nurse in each of the primary health clinics who were not only responsible for treatment of TB index patients, but also for contact tracing, evaluation of all household contacts, and provision of TPT to child contacts under 5 years of age. At the time of the trial and qualitative data collection, national guidelines for primary health care re-engineering mandated the use of ward-based outreach teams (WBOTs). Attached to a particular health facility and comprised of a professional nurse outreach team leader [10], approximately six community health workers (CHWs), a health promoter, and an environmental health practitioner, WBOTs were intended to provide a variety of community-based health services, including identifying and screening child TB contacts [12]. Though North West Province was quick to adopt this strategy, there was limited staffing or retention of dedicated nurse team leaders to manage the WBOTs and oversee the work of CHWs during the trial [13, 14].

At the time of the trial, child contact information was recorded onto the child’s road-to-health card held by the child’s caregiver. There were no clinic-based records for children without chronic illness at any of the 16 clinics. To follow child TB contacts over time, the trial therefore implemented a child contact record and register to track these children from identification through evaluation and eventual completion of TPT. These tools were designed, piloted and refined with nursing input prior to study initiation to improve acceptability and usability of the tools. The appearance of the child contact record and register were similar to the TB index patient file and register. Both consisted of clinically relevant check boxes that limited the amount of free text documentation. During the trial, data audits with individualized feedback were performed with each clinic’s TB nurse every 6 weeks for the initial 6 months and then quarterly for the duration of the 18-month study. These audit and feedback sessions aimed to enhance use of the clinical tools and identify barriers and potential solutions at each step of the pediatric TB prevention continuum of care. Finally, all nurses received initial training in either symptom-based or TST-based screening at the initiation of the study with ongoing supervision by a pediatric TB specialist that coincided with each audit and feedback session. Supportive supervision included feedback from chart reviews, answers to individual clinical questions, and review of the assigned screening algorithm to improve fidelity to the intervention.

Immediately post-trial, symptom-based screening was implemented as part of routine care in all Matlosana clinics. Interviews for this study occurred more than 6 months post-trial completion, therefore TB focal nurses in all 16 clinics who had participated in the trial, including those assigned to the intervention and control arms, had experience with symptom-based screening and all of the implementation strategies.

### Study population

We purposefully recruited focal TB nurses from all 16 trial-participating primary healthcare clinics and key healthcare administrators involved in TB and Maternal and Child Health programs for the Matlosana sub-district of North West Province, South Africa. 

### Procedures

A semi-structured topical field guide (Supplementary file 1) was developed to explore experience with the trial and perceptions of health system facilitators and barriers for successful provision of care and treatment for TB-exposed children less than 5 years of age. Development of the field guide was modeled on existing descriptive qualitative approaches to explore participant experiences with a trial, post-implementation [15–17]. Questions and case scenarios explored components of the intervention implemented during the trial, including: TB contact tracing, linkage to care, symptom screening, TPT initiation, and retention in care, as well as their suggestions about how to strengthen future interventions. Participants were also asked to reflect upon study implementation strategies including use of a new child contact record and register, ongoing supervision, and audit and feedback sessions that addressed both use of the newly implemented medical records tools and the continuum of care. All interviews were approximately one-hour and conducted one-on-one in English by female South African researchers trained in qualitative methods and interviewing techniques. Interviewers were not involved with the parent study and had no prior relationship with the participants.

### Analysis

All interviews were audio-recorded and transcribed. Thematic content analysis was used as the analytical approach [18]. A priori codes were initially developed based on the overall research question and interview guides and were used to direct the analysis. Emergent codes were created in response to identification and exploration of any unexpected domains during the coding process. The codebook was reviewed by two analysts before a final version was agreed upon. This integrated approach allowed for anticipated and unexpected patterns to emerge. The coding process was performed in parallel by two analysts who both had knowledge of the study and settings, but neither was part of the primary clinical trial. Coding was done individually and consensus on differing interpretations was achieved through discussion or settled by a third-party research team member, as needed. Coding of the transcripts was conducted using ATLAS.ti© analytical software. Codes were connected and categorized as part of the process to identify themes and were summarized using a template. The themes were continuously reviewed and discussed by the research team. Illustrative quotes are presented to represent participant views around each theme. Table 1 provides examples of codes used which assisted in the generation of each study theme. Given providers from both trial study arms had experience with the intervention and implementation strategies at the time of the interview, data were not separately analyzed by trial study arm. We ensured study rigor by establishing credibility through analyst triangulation and confirmability by ensuring that there was an audit trail of the analytical process and that the analysts practiced reflexivity during analysis [19]. Though sample size was determined by participation in the parent study, inductive thematic saturation was reached with no additional codes and themes emerging from the data [20]. Reporting of results adhered to the COREQ guidelines.

**Table 1:** Main study themes and examples of codes per thematic area. *indicates emergent code

Study Themes	Example of Study Codes
1. Nurse experiences with symptom-based TB screening: mixed opinions	• Challenges with symptom screening • Comfort using symptom screening • Challenges with tuberculin skin tests • Symptom screening process • Diagnostic tests used in the TB process • *Consequences of missing a case of TB • *TB patient mobility
2. Implementation experiences: willingness to adopt new clinical records and processes	• Positive descriptions of register • Positive descriptions of child contact record • Register use • Child contact record use • Plans to use child contact record in future
3. On-going challenges: barriers and solutions to optimizing screening of child TB contacts	• Challenges/barriers to screening • Health systems challenges for screening • Suggestions for job improvements to improve screening • Role of nurses in screening • Time management • Job demands that inhibit screening • Strategies to identify contacts • Which children get screened • *Consequences of missing a case of TB • *Nurse views on their role in identifying and screening

### Ethical considerations

All participants received oral and written information about the purpose of the study, risks and benefits, and voluntary participation including their right to withdraw from the study at any time for any reason. Data were anonymized, stored securely and presented to avoid identification of specific individuals. All study participants provided written informed consent prior to voluntary study participation and audio recording of the interview. This study was reviewed and approved by the University of Witwatersrand Human Research Ethics Committee and the Johns Hopkins Medicine Institutional Review Board.

## Results

### Participant characteristics

A total of 16 participants were interviewed for the study: 11 healthcare providers and 5 healthcare administrators. The overall median age for healthcare providers was 48 (IQR: 42–51) years but age was not collected for healthcare administrators. The median length of time that healthcare providers and administrators had worked in their posts was 15 (IQR: 4–29) and 8 (IQR: 8–11) years, respectively. All participants had a clinical background with majority being nurses (Table 2).

**Table 2:** Participant Demographics

	Health Care Provider *N* = 11	Healthcare Manager *N* = 5
Gender		
Female	9 (82%)	5 (100%)
Median Age (IQR)	48 years (42,51)	–
Training Background		
Nurse	11 (100%)	4 (80%)
Physician		1 (20%)
Other		
Median Length of Time at Current Post	15 years (4, 29)	8 years (8, 11)
Trial Clinic Assignment for Focal TB Nurses		
Symptom Clinic	5/11 (45%)	–
TST Clinic	6/11 (55%)	–

Overall, the primary findings outline healthcare providers and administrators’ varied opinions around symptom-based versus TST-based screening for child TB contacts and high acceptability of the trial implemented child contact record and audit and feedback system Additionally, participants discussed barriers and potential solutions to optimizing screening of child TB contacts with a focus on the supervision and training of CHWs and the role and responsibility of nurses for child TB care.

### Nurse experiences with symptom-based TB screening: mixed opinions

Healthcare providers had varied views when asked to reflect on their experiences with symptom-based screening (both during and after the trial). When compared to TST-based screening, symptom-based screening was deemed more favorable as it mitigated the delays in triaging and losses to follow-up that are associated with the skin test. “*Screening like that [symptom screening] means you have the child in front of you and you can do what is right for the child. For the TST, you know how many don’t even come back for the reading? So the child is here, and you can do what is necessary for the child”* (nurse, female). Furthermore, no additional resources were regarded as being necessary for performing a symptom screen, “*TST you need the equipment. If you don’t have TST it means you are not going to screen for that child. Yeah”* (nurse, female)*.*

Though symptom-based screening was perceived to be a simpler method, providers feared missing a symptomatic child. Nurses had limited pediatric history and physical exam skills and felt uncomfortable with the fact that young children are unable to directly communicate their symptoms to the provider, “*Symptom screening is easier [than TST], but you can miss … A patient, you can miss. Because now remember with symptom screening, we are relying on the mother”* (nurse, female). In particular, healthcare providers were concerned that caregivers might not disclose a child’s medical history, *“if the mother is openly enough there’s no way that you’re going to miss a child who’s sick*” (nurse, female). Additionally, providers were concerned that caregivers experience screening fatigue, “*I can tell you where a problem is already. The problem is, patients are not truthful. They will just say no, no, no, no, no, no, already knowing that if you say one yes, you’re going to go to the back and you’re going to be tested for TB. So some patients do that. Or they run out of the facility when people start screening. They do that, honestly, they do that. People do funny things.”* (nurse, female).

To deal with the subjective uncertainty of a clinical symptom screen, some providers in the symptom-based screening arm instituted an objective second test during the trial. Several providers noted using the TST as a confirmatory test after performing the symptom screen, “*I think both [symptom- and TST-based screening] are important because immediately, they can be-- I have those symptoms but I can’t confirm by just symptoms, there must be a test done. So immediately after you find out that [the child] presented this [symptoms], is whereby TST will confirm those tests, those symptoms of the child”* (nurse, female). Another provider described performing gastric aspirates on all household child contacts, irrespective of whether they had symptoms. “*I was doing everyone even if the screening tool says there are no signs there. After screening and there are no signs of TB I was doing the gastric washing just to be on the safe side, you see.” (nurse, female).* In general, providers feared the consequences of missing a sick child and in particular were worried about children dying as a result of not being diagnosed with TB disease “*The child is going to be more sick, and then is going to end up dying. And then, sometimes, the family thinks the child died because you couldn’t help the child. And when we hear that some child was here, and how he’s dead, ugh*” (nurse, male). Providers also blamed themselves if TB diagnoses were missed in children, again highlighting their desire to provide quality care to child contacts, *“it’s like you didn’t fulfill and then you didn’t do the right thing to that child” (*nurse, female).

### Implementation experiences: willingness to adopt new clinical records and processes

Willingness to adopt new clinical records and processes implemented under the parent study emerged as a key theme and facilitator to child TB contact management. Healthcare providers and administrators had positive perceptions of the child contact record and register and the audit and feedback sessions. These are important factors in the scale-up of symptom-based screening and child TB contact management.

### Child contact record

When discussing the child contact record, many providers considered it to be beneficial for patient care and easy to implement. The contact record provided a clinic-based method through which to track and manage patients through screening, evaluation, and their TPT course, allowing for identification of children lost to follow-up during the process, *“For the obvious reason that I said earlier. Like, it helped us in managing those children, like better than before, because like before it was chaotic. You will give the child isoniazid prophylaxis. The child will not come. After four months, the(y) come back, then you … -- you are not sure whether to start afresh …*” (nurse, male). The contact record was based at the clinic for nurses to manage and was also recognized as being easy to complete, as compared to the existing caregiver-managed, pediatric medical record, “- *we used to use the road to health chart. It’s a book. You have to go through the pages. [Healthcare providers] find that pink form its better, because like I said, [if there] are any symptoms, we have to screen the child every time he comes for treatment. [Pink file] It’s not that much. Unlike, we have to go through the book, looking for the page*.” (nurse, male). Additionally, providers utilized the child contact record and register to self-audit patient outcomes and manage follow-up appointments, “*Then you take your files, you sit, you check, oh, this one I haven’t seen. This one. Then you write your list. Already on Monday, you know gore (that) they must go and look for those that didn’t come.*” (nurse, female). Most healthcare providers described continuing to use the child contact record post-trial, further demonstrating its perceived usefulness.

### Child contact register

Providers reported that similar to the contact record, the register also served as a prompt tool for some nurses to ensure continued patient care, “*It reminds you of your patient, because you see so many patients every day, it’s easy to forget”* (nurse, female). However, post-trial feedback indicated that the register was less utilized than the contact record due to time constraints “*I was unable to use it. Yes, really, I was unable to use it. It’s still there. I tried to use it, but I didn’t have enough time to. I relied only on the pink form [child contact record]*.” (nurse, male).

### Audit and feedback sessions

In addition to provider self-audits, healthcare administrators favorably viewed the study-initiated audit and feedback sessions, where captured information was used to hold clinic staff accountable for patient outcomes “… *and the minute you do quarterly reviews you are shaking people to say Can you see what’s happening? Maybe you thought that you are doing what you’re supposed to do, but this is the proof that it’s not happening …”* (administrator, female). The child contact record and register were also recognized by providers as facilitating reporting mechanisms during feedback sessions by making additional information on IPT follow up available to the them, “*… especially with the TB indicators, it’s part of our IPT and we need to report on those, so it’s affecting our overall performance.*” (administrator, female).

Overall, participant feedback highlighted the willingness of healthcare providers to adopt new clinical records and processes within the clinic setting with the intention of improving child outcomes.

### On-going challenges: barriers and solutions to optimizing screening of child TB contacts

Several key themes related to roles and responsibilities within the health system emerged as barriers to effectively implementing or optimizing the pediatric TB screening process. These barriers are important in considering how a clinic-based screening intervention cannot improve TPT uptake without also taking in to account existing challenges within the health system.

### Challenges with utilizing community health workers

Healthcare providers discussed a number of their concerns with utilizing community health workers, responsible for much of the child contact tracing and linkage to care activities performed both in the clinic and in the community. Many of these concerns focused around CHWs identifying child contacts; a critical precursor to the success of clinic-based screening. A of lack training and supervision needed to perform their jobs satisfactorily was highlighted by most healthcare providers*. “Where was he [the CHW] when this particular person comes so ill in the facility … So where are they? Are they doing-- when they go, are they screening? Are they?” (*nurse, female*).* Many healthcare providers also discussed practical challenges to using CHWs for screening. *“I once discovered that the CHWs-- because they are assisting us with this [TB symptom screening] also-- I had to correct them, with some of the [screening] questions, that they didn’t really explain properly to the clients. It was just like a quick thing for them, but it was corrected. It’s [the screening tool] not problematic. It’s just that for them, it was a too quick thing. They just did it. Like [the question reads], “do you cough for two weeks?” “Do you sweat?” It’s just [they ask], “do you cough?” They don’t read the full question.”* (nurse, female). In line with concerns about the work of CHWs, some healthcare providers felt that CHWs required additional training, particularly related to identifying child contacts to ensure that they are able to be appropriately assessed at the clinic, either by TST or symptom screen. *“CHWs are our link [to patients]. We need to intensify their training.”* (nurse, female).

### Identifying and managing TB contacts – everyone’s responsibility?

Both healthcare providers and administrators stressed that for the benefit of child screening, all clinic staff should be competent in identifying and managing TB contacts, not just dedicated TB healthcare providers. If child TB contacts were to be appropriately managed, whether via TST or symptom screening, participants felt that: *“Everybody should know TB, not just the focal nurse. They all run away from TB.”* (nurse, female*).* In line with the need for increased competency in TB across all healthcare providers, some administrators discussed the negative consequences for child identification and contact tracing when responsibility for TB is siloed. *“The minute there is borders between programs people get lost at those borders.”* (administrator, female).

Despite the pervasive feeling across participants that everyone in the clinic should be competent in screening and managing child TB contacts, healthcare providers highlighted challenges when discussing their own experiences with integrated care and being expected to help out across the clinic. *“Usually, I help in front, when I’m busy pushing lines because we’re short staffed. I’ll be sitting in front doing chronics the whole day, or either I’ll be pulled to do ANC [antenatal care] or I’ll be pulled to do babies. Like, I’m always the one to have to fill holes. So it takes me away from TB quite a lot.”* (nurse, female).

### The scope of the nurse role

Most nurse healthcare providers expressed a desire to take on broader responsibilities for contact tracing and assessment of contacts outside of the clinic, particularly conducting household visits*.* The desire to take on additional responsibility in identifying child contacts revealed healthcare provider perceptions of gaps in the process of identifying child TB contacts and thus barriers to increased uptake of TPT. *“To go there [the household] and go and see for myself … see ourselves there are no under fives. Because you can say, the way we ask is, we might say at home, are there no what, what, what [children]? But you’ll find that the very same person is minding somebody’s child. When you come there [the household], there is a child.”* (nurse, female). This was also echoed by some health administrators *“I do not know if all the children actually come to the clinic at the end. So I think it would be better if we can take our bag with our [nasogastric] tubes and whatever and go and do the investigations there.” (*administrator, female). Most healthcare providers discussed how resources and demands on their time at the clinic limited activities like household visits that they perceived would improve child contact tracing and assessment. “*If you [nurses] can be offered cars and then enough personnel-- because sometimes you don’t do those house visits because when you leave the clinic you’re going to have more work. It’s just that you have to be full-time in the clinic, whereas you have to go in the community to go and check and look for the clients.”* (nurse, female).

## Discussion

This post-trial qualitative study provides important insight into provider and administrator experiences with the trial’s intervention, symptom-based screening, its implementation strategies including a TPT contact record, and key on-going challenges in the TPT continuum of care including child TB contact identification and linkage to care.. Participants reported high uptake and acceptability of trial implemented resources. Key health system issues included concerns about supervision and training of CHWs and the tension between nurses both wanting to take on more responsibility for child TB contacts and the requirement for TB to be managed uniformly by other health care workers across the clinic. Despite general approval of symptom-based screening and expressed benefits compared to TST, fears about missing a symptomatic child was a key reported challenge for implementation of clinic-based symptom screening guidelines. Understanding these challenges and how they hinder effective and optimized clinic-based screening and consequently the provision of child TPT is critical to developing responsive healthcare delivery models. Our findings highlight health system gaps that require attention to ensure effective improvements in the provision of child TPT and also demonstrate areas that may support TPT programs to find success.

First, healthcare providers are willing to adopt new clinic-based records and processes for child contact screening if they perceived them to be beneficial to patient care, easy to implement, and not time constraining. Existing literature from South Africa and other settings has documented nurse fatigue with excessive recordkeeping [21–23]. Furthermore, a recent systematic review examining child TB contact management in high burden settings found a lack of tools to support documentation was a common barrier to successful management of child TB contacts [11]. Our findings suggest the provision of simple tools that are designed with nurse buy-in may enhance uptake, and in this instance facilitate the provision of TPT. Additionally, audit and feedback sessions were deemed effective not only for monitoring statistics on patient outputs but also ensuring provider participation in the assessment of patient care activities [24]. The success of this approach highlights that nurses are receptive to supportive supervision and feedback, which has been previously noted as a deficiency in the South African healthcare human resources system [25, 26]. Both supportive supervision and engaging nurses with simple tools for documentation can be useful strategies in child TPT programs.

Second, providers in our study were apprehensive with the role that CHWs have in TB contact tracing and screening. Specifically, CHW’s quality of work is perceived as sub-optimal as a result of limited supervision and absence of training, supporting findings from two other recent studies of CHWs in South Africa [13, 27]. In response, nurses frequently expressed willingness to take on additional community-based TB work, despite also reporting they are regularly required to fulfill different clinical roles while no one else is willing or able to take on TB responsibilities. Nurses’ ability to engage with activities outside the clinic is limited due to competing demands as a result of facilities being under resourced and short staffed [26]. These factors also directly hinder nurses’ abilities to provide supervision to CHWs thus contributing to a lack of CHW oversight when working with TB patients. Nurses’ views on the failings of CHWs reflect key challenges pervasive in the Northwest Province’s implementation of WBOTs during the conduct of the trial and beyond, including poor recruitment and retention of supervisory team leaders and a pervasive lack of clarity on the scope and role of both CHWs and their nurse supervisors [13]. As South Africa continues to utilize CHWs at different steps in the child contact management cascade and use WBOTs as a strategy, attention must be paid to the ongoing implementation of this model and the existing deficits.

Third, despite proven efficacy of symptom-based screening for TB [28, 29], and extensive initial training and ongoing supportive supervision by a pediatrician for all participating nurses, there were concerns about missing a sick child. On one extreme, our study included a healthcare provider who chose to perform gastric aspirates, an unnecessary invasive procedure, on all child contacts, including those who were asymptomatic. While we await better diagnostic testing, symptom screening will remain an important tool to distinguish children who may need TB treatment from those who can safely start preventive therapy. Our clinical trial showed that training and supportive supervision provided skills necessary for healthcare providers to conduct symptom-based screening for child TB contacts with good clinical outcomes [10]. Our results in this report suggest that there is a need for additional training and supervision of healthcare providers, specifically addressing their concerns about relying on clinical evaluation alone to rule out TB disease and ensure successful TPT programs. This training should not only be focused on TB, but should also address providers’ other identified deficiencies in pediatric primary care and pediatric HIV care and prevention. Much of this can be offered through additional supportive supervision and mentoring of healthcare providers so skills learned through programs like the integrated management of child illness are translated into clinical care and maintained over time [30–32].

### Limitations

Limitations of the current study include having to conduct the interviews in English which potentially restricted the participant’s full description of their experiences. At the time of the interviews, all participants had been exposed to symptom screening and TST at their clinics with no differences in preferences across specific sites. As the interviews were conducted after the completion of the parent study, some healthcare providers who had participated in the clinical trial were not available to participate, thereby limiting our ability to interview health care providers from all 16 clinics. These qualitative study findings were not intended to be generalizable, but we describe valuable insights that may be transferable to other low- and middle-income settings with high TB burdens.

## Conclusion

Our study showed that the move to symptom screening to evaluate child TB contacts as part of starting TPT is an acceptable strategy for providers, but additional pediatric training is needed to allow providers to feel more comfortable with their decision making. Engaging nurses with simple tools and supportive supervision appear to be useful approaches for program implementation. Future interventions to improve child contact management in South Africa will need to address ambiguities in the responsibilities and management of CHWs in the WBOT system, to ensure seamless identification and linking of child TB contacts to care.

## Supplementary Information

**Additional file 1.**

## Data Availability

De-identified textual data can be made available upon reasonable request to the corresponding author.

## Abbreviations

CHW: Community health workers
HIV: Human immunodeficiency virus
TB: Tuberculosis
TPT: Tuberculosis preventive treatment
TST: Tuberculin skin test
UNHLM: United Nations High Level Meeting
WBOT: Ward-based outreach team
