# Supplementary material for: Post-trial perceptions of a symptom-based TB screening intervention in South Africa: implementation insights and future directions for TB preventive healthcare services
Source: BMC Nurs. 2021 Feb 8;20:29. doi: 10.1186/s12912-021-00544-z (PMC7869510; doi:10.1186/s12912-021-00544-z)
Supplement: Supplementary file 1 — Additional file 1. [file 12912_2021_544_MOESM1_ESM.docx]

Qualitative Field Guides for

Defining Health-System Factors that Affect Successful Delivery of IPT to Child Contacts: A Qualitative Study

**Semi-structured, in-depth interviews with clinical TB nursing staff**

**Study Arm** *(circle)*: Symptom-based TST-Based

**Date of interview: // (DD/MM/YYYY) Time: :**

Individual Participant Background Information:

*[For each participant, Please document the following: please write information in or circle appropriate response. Please ask in participant in private.]*

Participant Gender: Male, Female *(circle)*

Participant Age: _______

Participant Job Title: _________________________

Participant Training: Professional Nurse, Enrolled Nurse, Enrolled Nursing Assistant, Other *(circle)*

If Other: ______________________

How long have you been a (Professional Nurse/Enrolled Nurse/other Role)? _______________

How long have you worked in this clinic? __________________

How many years have you worked as a focal TB nurse in this clinic and/or other clinics?_______

*Open-ended questions:*

*Thank you for taking the time to speak with us today. As we discussed we are interested in how the process of TB screening, diagnosis and preventive treatment among CHILD TB CONTACTS is working in your clinic.*

*There are no right or wrong answers; I am here to learn from you. You may stop the interview at any time and you may choose to not answer any question you would prefer not to answer.*

*Do you have any questions before we start?*

*First, we want to talk about the screening process for child contacts of TB index cases who are under 5 years old at your clinic.*

1. Please tell me how the TB screening process for child TB contacts under 5 works at your clinic?

Probe: If intervention arm, probe on symptom-based screening

Probe: If control arm, probe on TST-based screening

Probe: When is the TST set? When is the TST read?

1. Tell me what happens when a child screens positive for TB?

Probe: Where do you refer children who screen positive? Is that always true for all children who screen positive?

Probe: What additional tests might be performed after a child screens positive?

Probe: Who should diagnose a child under 5 with TB?

Probe: What would make you think a child under 5 has TB?

3. What is working well about the current screening process at your clinic?

4. With regard to the screening process specifically (symptom or TST), what problems have you encountered?

*TST Clinics*:

Probe: What do you do when the mantoux is out of stock?

Probe: What do you do when the child comes late for a TST reading?

Probe: Has TST been a barrier to starting kids on IPT? Do many children have a TST placed, but never return to have it read?

*Symptom Clinics:*

Probe: How do you tell the difference between flu/cold (viral illness) and TB in a small child? How can you tell them apart?

Probe: How comfortable do you feel identifying sick children? How worried are you that you might miss a sick child? What are the consequences of missing a sick child?

5. What recommendations do you have to change the screening process?

6. The screening project introduced a pink file to help follow child contacts. How useful did you find this tool?

Probe: What did you find most useful?

Probe: What recommendations do you have to change the tool?

Probe: Was the file easy to read? (layout, font size, etc.)

Probe: Did the file contain too much or too little information? If you could add or

subtract something, what would that be?

Probe: Will you continue to use it after the project is completed?

7. The screening project introduced a child contact log to help follow child contacts. How useful did you find this tool?

Probe: If you could change some aspect of the log book, what would you change?

Will you continue to use it after the project is completed? Would you

recommend it to other clinics?

*Now I would like to ask you about how the IPT process is working for children contacts under 5 years old in your clinic from contact tracing through completion of IPT.*

8. How is the contact tracing process going for child contacts <5 in your clinic?

Probe: How do you identify child contacts? Do you think you identify all of the child contacts <5? Why or why not?

Probe: Do all child contacts under 5 return to the clinic for screening? What happens if they do not return to the clinic for screening?

Probe: Have any of these child contacts developed TB disease and been admitted to the clinic as index cases?

9. How do you calculate and prescribe pediatric doses? How difficult has this been for you and the rest of the TB staff?

10. What happens when the pediatric INH is out of stock? And when the pyridoxine is out of stock?

Probe: have stock outs of pediatric INH or pyridoxine been a challenge for the clinic?

11a. What other challenges have you faced in linking child contacts to care?

Probe: What challenges have you faced in identifying child contacts under 5 years?

Probe: What challenges have you faced linking these identified children into care such that they get screened and start IPT?

11b. What challenges have you faced in retaining child contacts in care for a full six months of IPT?

12. Tell me more about these challenges?

[Probe specifically on all issues mentioned]

13. How have you worked to resolve these different challenges at your clinic?

[Probe specifically on all issues mentioned]

14. How would you like to see this process work in the future?

15. What do you think can be done to help link more children to preventive TB services?

Probe: What can you do?

Probe: What can the clinic do?

Probe: What can the administration at West End do?

16. How do you view your role in the health system? What demands do you have on your time?

17. If you were asked by a close friend or relative whose child was a close contact of someone with TB, what would you recommend that they do?

Probe: Why should they bring or not bring their child to clinic for evaluation?

Probe: (Assuming the child screens negative) Why should they start or not start IPT?

Probe: Why should they finish or not finish IPT?

*We will present some scenarios below and we would like to know how you might handle these situations. Remember there are no right or wrong answers.*

16. (Symptom group only) You are reviewing a child contact of a TB index case. The mother reports that the child is completely well and denies all symptoms listed on the symptom screen. However, you notice the child is very tired and is clinging to mom. On the one hand, you think this child may just be fearful of medical professionals. On the other hand, you worry the child may be ill. What would you do in this situation?

16. (TST group only) You are reviewing a child contact of a TB index case. The mother reports that her child is completely well. She returns today, two days after the TST was placed and there is no induration (location of injection site is flat). However, you notice the child is very tired and is clinging to mom. On the one hand, you think this child may just be fearful of medical professionals. On the other hand, you worry the child may be ill. What would you do in this situation?

17. A DOTS worker/supporter identifies an infant living in the home of a TB index case that they was not reported to the TB nurse during the contact tracing process. You have discussed the situation with the index case and you learn that he only recently disclosed his TB status to his family. His niece and her child have been told they need to come to clinic, but have not yet come to the clinic for screening. What would you do in this situation?

18. Grandpa is diagnosed with TB. Mom lives and works in Johannesburg. The grandparents bring the child to the clinic but do not feel they can consent for either preventive treatment or HIV testing. They do not know the HIV status of their daughter. On the road to health card you see the child was tested at 6 weeks with a PCR. The child is now 18 months old. What are the issues and how do you proceed?

*Thank you for your time. We appreciate all your inputs and suggestions. Is there anything else you would like to add?*

End time: :

**Key-Informant Interview Guide**

**(District and Sub-district Program Managers, etc)**

**Job Title: ____________________________________________**

**Date of interview: // (DD/MM/YYYY) Time: :**

**Participant Sex: Male Female (circle)**

**INTRODUCTION:**

*Thank you for taking the time to speak with us today. As we discussed we are interested in how the process of TB screening, diagnosis and preventive treatment among CHILD TB CONTACTS is working in the Matlosana sub-district.*

*There are no right or wrong answers; I am here to learn from you. You may stop the interview at any time and you may choose to not answer any question you would prefer not to answer.*

*Do you have any questions before we start?*

***Background information:***

*First, I would like to start by asking you some questions about your background.*

[Please write information in or circle appropriate response]

**D1. Participant Job Title:** _________________________

**D2: Participant Training:** *(circle)*

Doctor Enrolled Nurse

PHC Nurse Other: ______________________

Professional Nurse

**D3. How long have you been a (Doctor/Nurse/etc)?**__________________

**D4. How long have you worked in this role (D1)?** __________________

***Open-ended questions:***

1. Please tell me a bit about your role in the health care system.

Probe: What is your role with regard to outpatient health services?

Probe: What is your role with regard to TB services?

Probe: What is your role with regard to child health services?

***Now I would like to ask you some questions about TB screening in Matlosana.***

*We are going to talk about the screening process for children who are close contacts of anyone who has been diagnosed with TB. As you know, this screening process takes place in the clinic by the focal TB nurses. During our study, half of the clinics continued to do TST-based screening and the other half implemented the new guidelines and performed symptom-based screening. In either case, if the child screens negative they are started on isoniazid preventive therapy and if they screen positive, they are evaluated by a physician either at the clinic or at Klerksdorp Hospital. They decide whether a child is started on preventive therapy or tuberculosis treatment.*

1. How did you first learn about this project?
2. What is your understanding of how this program fits into the current pediatric tuberculosis guidelines?
3. How have you and your team been engaged in the project?

Probe: Was this sufficient or do you wish you had been engaged more?

Probe: Should anyone else have been engaged in the conversation?

1. How did you receive updates on the project?

Probe: Was this sufficient? How else could PHRU have engaged you in updates?

1. How successful do you feel the project has been?

Probe: How could PHRU have implemented this project differently to ensure more success?

1. How do you think the health care system could be improved to help identify and place more children on IPT?

Probe:

To improve contact tracing or identification of the child contacts

To improve linkage of the identified child with TB preventive care

To improve retention in care and completion of TB preventive treatment?

1. Household based contact tracing (identifying children and others at risk in the TB index case’s home) identifies more children than contact tracing in the clinic. Nurses report a number of challenges including index cases misrepresenting where they live, their contact information, who lives in their households, and whether they will allow CHWs to visit their home. How do you think we can improve the health care system to help the clinic nurses solve this/these problem(s)?
2. About 50% of child contacts are never brought to the clinic for evaluation/screening for TB. In order to eliminate childhood TB from Matlosana, it is recommended to link more than 90% of child contacts with TB preventive care. How do you think the health care system could improve this situation / or address the challenges to help the clinic nurses?

Probe: How could integration of TB into maternal and child health services facilitate this?

1. How important do you think it is to maintain the child contact evaluation program including formalized child contact record keeping and presentation of quarterly indicators, as we have been doing, in order to continue to assess child health within the TB program even after the study is completed?

Probe: What additional resources might be necessary to achieve this? Is the sub-district TB program able to accomplish this?

1. What would it take to expand symptom-based screening and formalized child contact record keeping to clinics outside of Matlosana?

Probes: What personnel would you need? What resources would you need?

What types of policy direction and guidance would you need to be in place?

How do you think the current study could help you move in that direction?

In what ways could this type of study be more helpful in the future to that end?

1. Have you considered performance-based incentives or other accountability mechanisms to maximize productivity in the clinic? How do you think these could be implemented to be effective?
2. Clinics report that there continue to be a number of challenges in HIV-testing vulnerable children whose mothers have left them with grandparents or other family while they work full time in other locations. These family members do not feel they are able to provide consent for the child’s HIV testing or TB preventive treatment. Clinic nurses do not feel the laws in South Africa allow them to HIV test the child after six weeks of age. How can this problem best be addressed?
3. As you know, stigma continues to affect how patients seek medical care. Nurses report many patients attend clinics in areas where they are not assigned. Is this something the health care system should be accommodating? How can this problem best be addressed?

*Thank you for your time. We appreciate all your inputs and suggestions. Is there anything else you would like to add?*

End time: :
